# Highly flexible, foldable, and rollable microsupercapacitors on an ultrathin polyimide substrate with high power density

**DOI:** 10.1038/s41378-018-0016-3

**Published:** 2018-07-30

**Authors:** Juan Pu, Xiaohong Wang, Renxiao Xu, Sixing Xu, Kyriakos Komvopoulos

**Affiliations:** 1 Department of Microelectronics and Nanoelectronics, Tsinghua University, Beijing, 100084 China; 2 Institute of Microelectronics, Tsinghua University, Beijing, 100084 China; 3 Tsinghua National Laboratory for Information Science and Technology, Beijing, 100084 China; 4 Department of Mechanical Engineering, University of California, Berkeley, CA 94720 USA

## Abstract

The design and functionality of extremely flexible, foldable, and rollable microsupercapacitors (MSCs) with in-plane interdigital electrodes that consist of single-walled carbon nanotube (SWCNT) networks on an ultrathin polyimide substrate are demonstrated through experiments and finite element simulations. The all-solid-state MSCs can be reversibly bent, folded, and rolled purely elastically without degradation of their electrical performance. The simulation results confirm that the deformation in bent, folded, and rolled MSCs is purely elastic. The high power density (1125 W cm^–3^) and small time constant (1 ms) of the present MSCs are comparable to those of aluminum electrolytic capacitors. The MSCs operate at scan rates of up to 1000 V s^–1^, are characterized by a volumetric capacitance of 18 F cm^–3^ and an energy density of 1.6 mWh cm^–3^, and exhibit superior electrochemical stability with 96% capacity retention even after 100,000 charge/discharge cycles. The developed MSCs demonstrate high potential for integration in flexible and wearable electronic systems.

## Miniturized electronics: Highly flexible and high-power microsupercapacitors on ultrathin substrates

The rapid growth of next-generation stretchable and flexible technologies suggests critical needs for developing tiny energy storage devices with corresponding mechanical properties. Researchers from China and the United States, led by Xiaohong Wang of Tsinghua University, and the University of California’s Kyriakos Komvopoulos, discovered a scalable and simple way to produce a high-performance version of the devices, called microsupercapacitors (MSCs). The flexible, foldable, and rollable MSCs are produced through a one-step lift-off process of the sprayed carbon nanotubes films on ultrathin substrates. The MSCs outperform most of the existing, similar technologies, and offer performance indices comparable to classic aluminum electrolytic capacitors. As the devices retain 96% of their original capacity after 100,000 cycles, it is suggested that they may be well-suited for long-term applications such as in wearable technology that harvests energy from its wearer.

## Introduction

Electric double-layer capacitors (EDLCs), also known as supercapacitors or ultracapacitors, rely on the rapid and reversible adsorption/desorption of ions at the electrode–electrolyte interface for charge storage. This kind of charge storage mechanism offers EDLCs several desirable properties including long operation life (>10,000 cycles) and high power density. Typically, EDLCs deliver a power density about an order of magnitude higher than that of lithium-ion batteries and an energy density two orders of magnitude higher than that of electrolytic capacitors. Therefore, EDLCs could potentially replace traditional electrolytic capacitors to enable the further size reduction of electronic circuits.The highlighted surnames are correct.

The rapid growth of microscale flexible electronics such as sensitive robotic skin^1^, wearable photovoltaics^2^, flexible transient electronics^3^, and finger-tip devices^4^ has increased the demand for microscale flexible energy-storage units, such as microsupercapacitors (MSCs). The MSCs with two-dimensional (2D) in-plane electrode structures have a lower device thickness than conventional supercapacitors with sandwiched electrodes, which not only endows the device with greater flexibility, but also provides the potential to be folded and rolled to adapt to more applications. Moreover, because the ionic diffusion paths in 2D in-plane electrodes are much shorter, the rate capability and power performance of the devices can be greatly enhanced^5–7^. These properties are particularly important when the MSCs are coupled with microbatteries, microfuel cells, and energy harvesters to provide maximum power or when they are used to replace electrolytic capacitors in applications such as filtering voltage ripples in line-powered electronics (ac line filtering)^8,9^.

Significant effort has focused on the design and fabrication of highly flexible and conductive electrodes with interconnected micro/nanostructures, such as carbide-derived carbon^10,11^, carbon nanotubes (CNT)^12,13^, graphene^14–16^, and graphene/CNT carpet^17^. Single-walled CNT (SWCNT) networks provide significantly higher conductivity because the few interparticle contact yields a lower-contact resistance than most other carbonaceous materials^18^ used as electrode materials for high-power MSCs. Moreover, CNT networks show superior robustness under bending, abrasion, and stretching, and their functionality is not affected by the development of mechanical stresses^19–23^. In addition, the networks generally offer a high fault tolerance because many different current paths exist even with the presence of a few disconnected or missing links in the network. Various methods have been successfully developed to fabricate CNT networks for energy-storage applications including vacuum filtration to form thick, free-standing membranes^24,25^, mixing with a binder material^26,27^, and electrophoretic deposition^13^. Although these methods can bring excellent electrochemical performance, wearable electronics impose increasing demands for energy-storage units with high-power behavior, as well as foldability, rollability, and stretchability. Dim et al. achieved stretchable MSC arrays with in-plane SWCNT electrodes using spray deposition^28^. The device showed stability under stretching and bending. We also previously reported stretchable MSC arrays with in-plane SWCNT electrodes fabricated on honeycomb polydimethylsiloxane (PDMS) substrates that exhibited good capacitive performance and excellent rate capability^29^.

The objective of this study is to present a facile and scalable method of fabricating in-plane interdigital electrodes consisting of SWCNT networks by combining a simple spray-deposition technique with a single-step lift-off process. The produced SWCNT network is ultrathin, extremely flexible, and completely accessible by electrolyte ions during charging and discharging. The high flexibility and surface mountability of the present SWCNT MSCs are largely due to the ultrathin (~1.3-µm thick) polyimide (PI) substrate. Because of the high electrical conductivity of spray-coated SWCNT networks and the small space (40 µm) between the in-plane interdigital electrodes, the developed all-solid-state MSCs can operate at a high scan rate of 1000 V s^–1^ and demonstrate a volumetric capacitance of 18 F cm^–3^, a maximum energy density of 1.6 mWh cm^–3^, and a maximum power density of 1125 W cm^–3^. Moreover, the present microdevices exhibit superior electrochemical stability (i.e., 96% capacity after 100,000 charge/discharge cycles), exceptional flexibility, and insignificant degradation in electrical performance even after excessive bending, folding, or rolling.

## Materials and methods

### Spray deposition of SWCNT electrodes

Purified SWCNTs (P3-SWNT, Carbon Solutions) with 1–3% carboxylic acid-surface functional groups were used as electrode materials. The SWCNTs were dispersed in deionized (DI) water with a tip sonicator for 1–2 h to form a 0.5–1 mg/ml stable suspension. The suspension was then sprayed onto a Si(100) wafer and placed onto a hotplate heated to 40–60 °C to form ~140-nm-thick SWCNT films. The sprayed SWCNT films were used as MSC electrodes without further treatment.

### Preparation of gel electrolyte

The polymer electrolyte (PE) was prepared by mixing 10 ml of DI water with 10 ml of phosphoric acid (H_3_PO_4_) under magnetic stirring for 30 min and, separately, dissolving 10 g of polyvinyl alcohol (PVA) in 90 ml of DI water at 90 °C under magnetic stirring for 1 h. Finally, the two solutions were mixed under magnetic stirring for 1 h. The PVA:H_3_PO_4_ ratio in PE was fixed at 1:1 wt/vol.

### Fabrication of flexible SWCNT MSCs

MSCs with SWCNT electrodes were first fabricated on a Si(100) wafer by standard microfabrication techniques and, subsequently, carefully peeled off to obtain free-standing flexible MSCs. The first step of the fabrication process was to spin-coat a PI layer onto a Si(100) wafer (Supplementary Information, Fig. S1a). The PI solution (ZKPI-306II, POME Sci-tech, Beijing, China) was mixed with a PI thinner (POME Sci-tech, Beijing, China) in a weight ratio of 5:1 to form a diluted PI solution, and then left overnight to allow the bubbles in the diluted PI solution to escape. Then, the diluted PI solution was spin-coated onto a Si(100) wafer in two steps (step 1: 800 rpm for 18 s and step 2: 6000 rpm for 60 s), soft baked in an oven at 80 °C for ~3 h for the solvent to evaporate, and cured in the oven at 250 °C for ~2 h. The PI film thickness was found to be ~1.3 µm. A bilayer consisting of an ~10-nm-thick Cr underlayer and an ~130-nm-thick Au top layer was evaporated onto the Si(100) wafer and patterned using a lift-off process to form the interdigital current collectors and contact pads of the MSCs (Fig. S1b). The lift-off process was performed in acetone under very gentle sonication. The SWCNTs were then spray-deposited onto the electrode area and patterned to interdigital electrodes by a lift-off process (Fig. S1c). The interdigital pattern of the SWCNT layer was the same as that of the underlying Cr/Au bilayer. For the lift-off process of the Cr/Au bilayer or the SWCNT layer, two layers of a photoresist film were spin-coated onto the substrates. The first photoresist layer (AR-P 5480, 100:50 dilution, Microchemicals) was spin-coated at 700 rpm for 9 s and at 2000 rpm for 40 s, and then soft baked at 170 °C for 5 min. The second photoresist layer (AZ 601, Microchemicals) was spin-coated at 700 rpm for 9 s and at 3000 rpm for 40 s, and then soft baked at 100 °C for 2 min. Finally, the two layers of the photoresist were exposed to UV light and developed. The fabricated devices were carefully peeled off the Si substrate (Fig. S1d) using the methods shown schematically in Fig. S2. Then, the PVA-H_3_PO_4_ gel electrolyte was coated onto the interdigital electrodes and left overnight at room temperature for the excess water to evaporate (Fig. S1e). Finally, a 10-µm to 20-µm-thick layer of PDMS was applied to encapsulate the microdevices (Fig. S1f). The PDMS resin and curing agent (10:1 weight ratio) were mixed for 5 min, degassed in vacuum for ~30 min, carefully placed on the device surface, and finally cured at room temperature for ~24 h.

### Characterization techniques

The microstructure of the sprayed SWCNTs was examined with a scanning electron microscope (SEM) (S-5500, Hitachi, Tokyo, Japan). For cross-sectional SEM imaging, the microdevices were sectioned perpendicular to the finger length direction and coated with an ~1-nm-thick Au-Pd layer to enhance the surface conductance. To prepare the samples for transmission electron microscope (TEM) imaging, the SWCNT powder was dispersed in DI water with a tip sonicator for ~2 h to form a 0.05 mg/ml stable suspension. Finally, a drop of the prepared suspension was applied to a standard Cu grid for imaging with the TEM (JEOL JEM2011, Peabody, Massachusetts, USA).

### Electrochemical testing

The electrochemical performance of the SWCNT MSCs with the PVA-H_3_PO_4_ gel electrolyte was evaluated with a two-electrode system. Cyclic voltammograms (CV) and galvanostatic charge/discharge (GCD) experiments were performed with a CHI 860D electrochemical workstation. In the CV tests, the scan rate and voltage were varied in the range of 0.5–1000 V s^–1^ and 0–0.8 V, respectively. In the GCD experiments, the microdevices were charged and discharged using a charge/discharge current of 10 nA cm^–2^ and applying a voltage in the range of 0–0.8 V.

The volumetric capacitance *C*_*v*_ was determined from the CV response for scan rates in the range of 0.5–1000 V s^–1^ using the relation.

1 $$C_v = \frac{1}{{{\cal V}\Delta V}}\mathop {\int }\nolimits I(t){\mathrm{d}}t$$

where Δ*V* is the potential range (=0.8 V), ${\cal V}$ is the total electrode volume (including the volume of the electrodes and interspace), *I*(*t*) is the current measured during CV testing, and *t* is the time.

To determine the capacitance of the SWCNT layer, the capacitance of the Au current collector was subtracted from the total capacitance of the Au + SWCNT layers. In this paper, the MSC capacitance refers to that contributed only by the SWCNT electrodes.

The volumetric energy density *E*_V_ and power density *P*_V_ were calculated from the CV responses for a scan rate in the range of 0.5–1000 V s^–1^ using the relations

2 $$E_{\mathrm {V}} = \frac{1}{2}C_{\mathrm {s}}\Delta V^2/3600,$$

3 $$P_{\mathrm {V}} = \frac{1}{2}\frac{{C_{\mathrm {s}}\Delta V^2}}{{t_{\mathrm {d}}}},$$

where *t*_d_ (in seconds) is the discharge time. The energy and power densities are those contributed by the SWCNT electrodes.

Electrochemical impedance spectroscopy (EIS) was performed by applying a 10 mV ac signal over the frequency range of 1–10^6^ Hz using an impedance/gain-phase analyzer (Solartron 1260, AMETEK Advanced Measurement Technology, Farnborough, Hampshire, UK). The real and imaginary parts of the impedance, *Z*' and *Z*'', respectively, were recorded over the whole-frequency range and plotted as a Nyquist plot. The real and imaginary parts of the capacitance, *C*' and *C*'', respectively, were obtained by

4 $$C^\prime = \frac{1}{2}\frac{{ - Z\prime }}{{\pi f\left| Z \right|^2}}$$

5 $$C\prime\prime = \frac{1}{2}\frac{{Z\prime\prime }}{{\pi f\left| Z \right|^2}}$$

where $f$ is the frequency and $\left| Z \right| = \sqrt {Z\prime ^2 + Z\prime\prime ^2}$ is the absolute value of the impedance.

To investigate the electrochemical stability of the microdevices, cyclic CV tests were performed over the potential range of 0–0.8 V at a scan rate of 50 V s^–1^. The MSCs were subjected to a total of 100,000 charge/discharge cycles. To evaluate the electrochemical stability of the microdevices during bending, folding, and rolling, CV curves were recorded over the potential range of 0–0.8 V at a scan rate of 10 V s^–1^.

### Finite element analysis (FEA)

A three-dimensional FEA was performed with the multi-physics code ABAQUS to elucidate the microdevice deformation under various loading conditions including wrapping around rods of varying radii, folding, and rolling. In each FEA model, the elastomer (i.e., PDMS) and the multilayer circuit (i.e., Au/PI) were modeled with eight-node, hexahedral, brick, solid elements (C3D8R) and quadrilateral shell elements (S4R), respectively. The very thin CNT and Cr layers were not included in the FEA model because their effect on the microdevice mechanics is negligible. In view of the good adhesion of the microdevice materials, perfect interfacial bonding was assumed in all FEA simulations. Refined meshes were used to ensure accurate strain mapping. The Mooney–Rivlin model was used to simulate the behavior of PDMS with an effective elastic modulus of 1.1 MPa and Poisson’s ratio of 0.49. The PI layer was modeled as a linear elastic material with an elastic modulus of 2.5 GPa and Poisson’s ratio of 0.34. The Au layer was modeled as an elastic perfectly plastic material with an elastic modulus of 78 GPa, Poisson’s ratio of 0.44, and yield strength of 234 MPa.

## Results and discussion

**Fig. 1:**
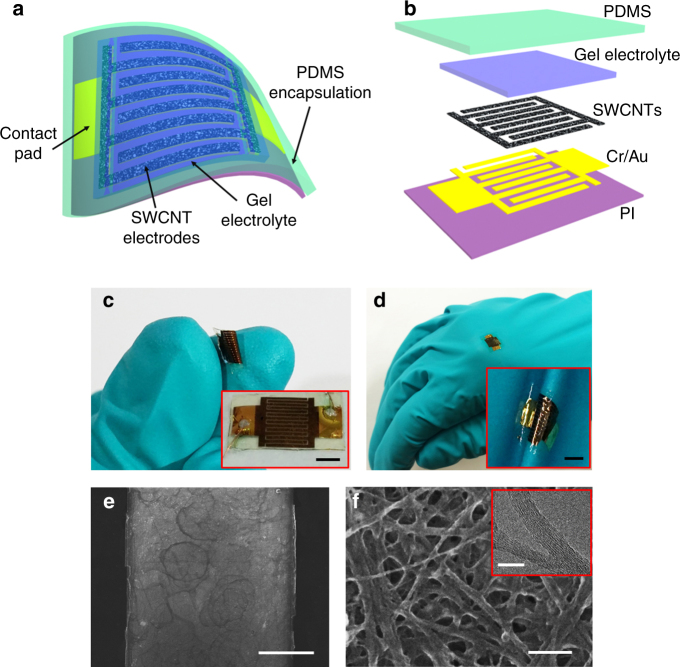
Flexible, surface mountable MSCs and SWCNT electrode microstructure: **a** schematic of a flexible SWCNT MSC on an ultrathin PI substrate; **b** exploded view of the MSC layered structure; **c** digital photograph of a SWCNT MSC bent between two fingers (the inset shows the undeformed device; scale bar = 2 mm); **d** digital photograph of a SWCNT MSC mounted on a latex glove (the inset shows the microdevice conformably mounted onto the raggedy glove surface; scale bar = 2 mm); **e** top-view SEM image of the SWCNT electrode; and **f** high-magnification SEM image of the spray-deposited SWCNT network (the inset shows a TEM image of SWCNTs). The scale bars in (**e**, **f**) and inset in (**f**) are 100 µm, 100 and 20 nm, respectively

The SWCNT MSCs were fabricated on an ultrathin PI substrate to obtain highly flexible and easily mountable devices on various surfaces and, in turn, enable their application in flexible and wearable electronics. Schematic illustrations of a flexible SWCNT MSC fabricated on an ultrathin PI substrate and its layered structure are shown in Figure 1a, b, respectively. Conventional lithography and mechanical peel-off techniques were combined to fabricate all-solid-state MSCs with in-plane interdigital SWCNT electrodes on free-standing ultrathin PI substrates.

Figure 1c, d shows a fabricated microdevice bent between two fingers and mounted on a latex glove, respectively. The inset of Fig. 1c shows the undeformed microdevice, whereas the inset of Fig. 1d illustrates the conformal attachment of the microdevice onto the rugged glove surface. Optical microscope images obtained before peeling-off the microdevice from the PI/Si substrate showed that the width of the interdigital fingers is 300 µm, while the space between the fingers is 40 µm (Fig. S3). Figure 1e, f shows low-magnification and high-magnification SEM images of SWCNT electrodes, respectively. The ring-like patterns on the SWCNT electrodes (Fig. 1e) occurred during drying of the sprayed tiny droplets of SWCNT aqueous solution. The spray-deposition process produces a random network of entangled SWCNTs (Fig. 1f). The high current capacity and mechanical strength of the SWCNT networks are critical factors for the robustness and flexibility of the microdevices. The TEM image shown in the inset of Fig. 1f reveals that the diameter of a SWCNT bundle is in the range of 10–30 nm. A comparison of the cross-sectional SEM images of the Cr/Au + SWCNT layer (Fig. S4a) and the Cr/Au layer (Fig. S4b) fabricated on a PI layer indicates that the thickness of the SWCNT layer on the Cr/Au bilayer is ~140 nm.

Raman and Fourier transform infrared (FTIR) spectroscopy were used to examine the molecular and chemical structure of the SWCNTs, respectively. The very low D-to-G band ratio (*I*_D_/*I*_G_ = 0.078) calculated from the Raman spectrum (Fig. S5a) reveals high-purity SWCNTs with a very low defect density^30,31^. The carboxylate –OH stretching peak centered at 3448 cm^–1^ in the FTIR spectrum (Fig. S5b) confirms the surface functionalization of the SWCNTs by the carboxylic acid groups^32^.

**Fig. 2:**
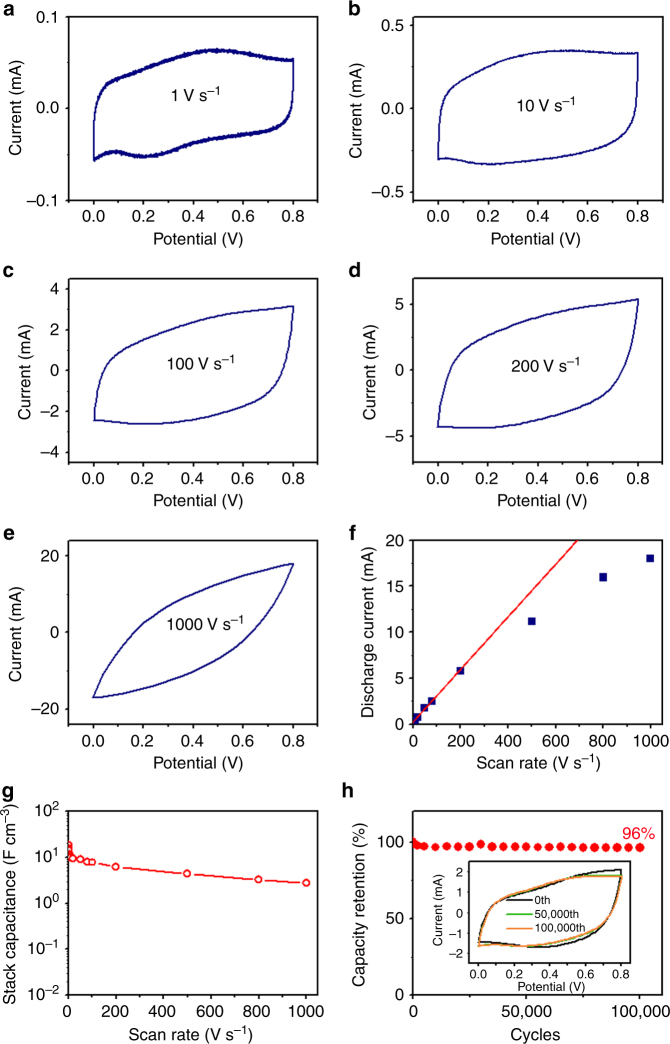
Electrochemical performance of SWCNT MSCs: **a**–**e** representative CV curves of SWCNT MSCs at scan rates of 1, 10, 100, 200, and 1000 V s^–1^, respectively, **f** discharge current vs. scan rate, **g** stack capacitance *C*_V_ vs*.* scan rate, and **h** capacity retention vs. electrochemical charge/discharge cycles at a scan rate of 50 V s^–1^ (the inset shows the CV curves corresponding to the 0th, 50000th, and 100000th cycle)

To evaluate the electrochemical performance of the SWCNT MSCs in terms of the capacitance and power capability, CV testing was performed at scan rates of 0.5–1000 V s^–1^. For scan rates of 1–200 V s^–1^, the rectangular-like shape of the CV curves (Fig. 2a–d) suggests the formation of an efficient EDLC and the occurrence of a fast-electrode charging process. At extremely high scan rates (e.g., 1000 V s^–1^), the shape of the CV curve became quasi-rectangular (Fig. 2e). A linear discharge current response was observed up to a scan rate of 200 V s^–1^ (Fig. 2f), which is indicative of high rate capability. The SWCNT MSCs of this study outperform most reported MSCs in terms of the formation of the perfect electric double layer and a high power capacity (rate capability)^28,33–37^. In general, the smaller the distance between the interdigital electrodes, the higher the power capability of the microdevice, because of the significant decrease in the mean ion diffusion path between electrodes. The narrowness of the gap between the interdigital electrodes is usually limited by the fabrication technique. A very small gap (40 µm) between the spray-deposited SWCNT electrodes with a high yield was achieved with the bilayer lift-off process (Fig. S6).

At a low scan rate of 0.5 V s^–1^, the volumetric capacitance of the microdevice is equal to 18.1 F cm^–3^, and decreases to 10 F cm^–3^ with an increase in the scan rate to 10 V s^–1^ (Fig. 2g), i.e., 55% capacitance retention. Increasing the scan rate to 100 V s^–1^ decreases the volumetric capacitance to 7.7 F cm^–3^, which represents 43 and 77% of the volumetric capacitance at scan rates of 0.5 and 10 V s^–1^, respectively. The all-solid-state microdevice exhibits a very high volumetric capacitance of 2.8 F cm^–3^, even at a very high scan rate of 1000 V s^–1^. The capacitance of the bare Au electrodes is less than that of the SWCNT electrodes by more than an order of magnitude (Fig. S7e). Thus, it may be interpreted that the microdevice capacitance is mainly due to ion transfer in the microstructure of the SWCNT network. As mentioned earlier, the present MSC capacitance is only due to the SWCNT electrodes. The electrochemical properties of the SWCNT MSCs can be further elucidated by comparing their performance with those of other MSCs. The volumetric capacitance of the SWCNT MSCs is comparable to those of most reported EDLC MSCs (0.2–20 F cm^–3^)^15,28,33–38^. Some other reported MSCs such as reduced graphene oxide MSCs^16^ and polyaniline nanowire MSCs^39^ demonstrate higher specific capacitance, but poorer rate capability and frequency response.

The GCD response of the SWCNT MSCs for a current density of 10 nA cm^–2^ is fairly symmetric, approximately triangular, and does not show an obvious IR drop, i.e., an abrupt voltage decrease at the onset of GCD discharging (Fig. S8). This is illustrative of the high Coulombic efficiency, fast charging across electrodes, and low equivalent series resistance (ESR) of the microdevices, in agreement with the CV results (Fig. 2a–g). The reproducible and stable capacitive behavior of the SWCNT MSCs with the PVA–H_3_PO_4_ solid-state electrolyte was observed up to 100,000 electrochemical charge/discharge cycles (Fig. 2h). Remarkably, the microdevices maintained 96% of their initial capacitance even after 100,000 cycles. This unprecedented electrochemical stability of the all-solid-state SWCNT MSCs is illustrative of their great potential for long-term, high-cycle applications such as energy storage for energy harvesting systems.

**Fig. 3:**
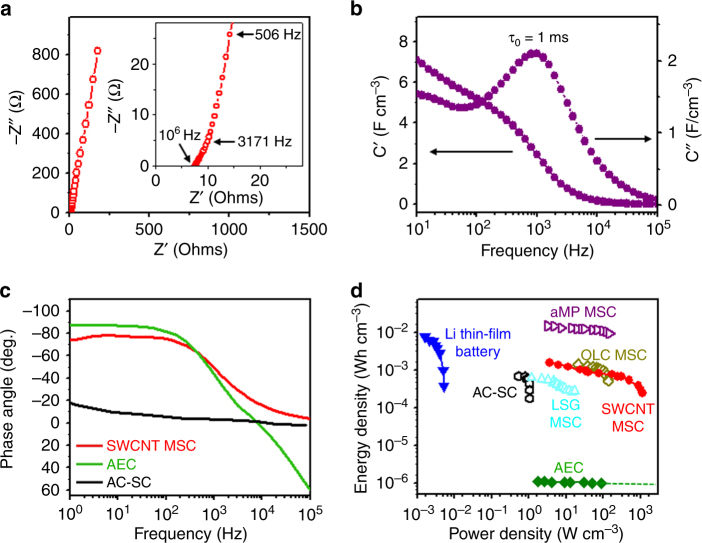
Frequency response, energy density, and power density of the SWCNT MSCs: **a** Nyquist plot (imaginary impedance *Z'* vs. real impedance *Z"*) with the magnified high-frequency region shown in the inset; **b** real and imaginary parts (*C'* and *C*", respectively) of the stack capacitance vs. frequency; **c** impedance phase angle vs. frequency of a SWCNT MSC, a commercial activated carbon supercapacitor (AC-SC)^37^ and an aluminum electrolytic capacitor (AEC);^37^ **d** Ragone plots (energy density vs. power density) of a SWCNT MSC, a commercial 4 V/500 µAh Li thin-film battery^31^, a 2.75 V/44 mF AC-SC^31^, a 3 V/300 µF AEC^31^, a LSG MSC^37^, an OLC MSC^6^, and an aMP MSC^36^

The superior power performance of the present microdevices was further demonstrated by the EIS results. An almost vertical linear response was found in the low-frequency region of the Nyquist plot of a SWCNT MSC (Fig. 3a), indicating nearly ideal capacitive performance. The absence of a semicircular response in the high-frequency region (see inset of Fig. 3a) is also illustrative of the ultrahigh ionic conductivity at the electrode/electrolyte interface^40^, which is consistent with the observed high rate capability and high power performance. The microdevice exhibits pure capacitive behavior even at a high frequency of 3171 Hz (see inset of Fig. 3a), attributed to the highly accessible surface of the SWCNT network. The ESR (estimated from the *x*-intercept in the Nyquist plot) of the current microdevice is ~7.5 Ω. The low ESR value is attributed to the highly conductive SWCNT network deposited onto the Cr/Au bilayer current collector, which produced a low internal and interfacial resistance, and the interconnected micropores of the SWCNT network that were completely accessible for ion adsorption/desorption.

For a more informative analysis of the EIS results, the frequency dependence of the real and imaginary parts of the volumetric capacitance, *C'* and *C"*, respectively, were examined (Fig. 3b). The fast frequency response of the SWCNT MSCs is further illustrated by the very small relaxation time constant *τ*_0_ = 1 ms, representing the minimum time for completely discharging the device with >50% efficiency^38,41^, determined from the peak frequency *f*_0_ of *C"* by *τ*_0_ = 1/*f*_0_ The relaxation time of the SWCNT MSCs is significantly shorter than that of conventional EDLC MSCs (*τ*_0_ ≈ 1 s) and most high-rate MSCs (*τ*_0_ = 3–30 ms)^35,37,41–44^, and comparable to that of an aluminum electrolytic capacitor (AEC; *τ*_0_ ≈ 1 ms). While electrochemically reduced graphene oxide MSCs^8^ and graphene/CNT carpet-based MSCs^17^ show similar *τ*_0_ values, they exhibit a lower specific capacitance than SWCNT MSCs. The impedance phase angle versus frequency plots of a SWCNT MSC, an activated carbon supercapacitor (AC-SC)^43^, and an AEC^43^ are shown in Fig. 3c. The phase angle of the SWCNT MSC reaches –45° at 1266 Hz, compared with ~1000 Hz for the AEC and ~0.15 Hz for the AC-SC, which is also indicative of the very small relaxation time constant of the SWCNT MSC.

The excellent performance of the SWCNT MSCs is also illustrated by the Ragone plots shown in Figure 3d. The energy and power density of different commercial energy-storage devices designed for power microelectronic applications, i.e., a 4 V/500 µAh Li thin-film battery^36^, 3 V/300 µF AEC^36^, 2.75 V/40 mF AC-SC^36^, laser-scribed graphene (LSG) MSC^43^, onion-like carbon (OLC) MSC^38^, and activated mesophase pitch (aMP) MSC^42^, are also shown for comparison. Remarkably, the SWCNT MSC shows a maximum energy density of 1.6 mWh cm^–3^, which is much higher than that of the AEC, LSG MSC, and AC-SC and comparable to that of the Li thin-film battery and OLC MSC. The maximum power density of 1125 W cm^–3^ of the SWCNT MSC is significantly higher than that reported for high-power performance MSCs such as OLC, LSG, and aMP MSCs and comparable to that of AEC (10^1^–10^3^ W cm^–3^)^36^. A detailed comparison of the SWCNT MSC with reported high-power MSCs can be found in Table S1.

**Fig. 4:**
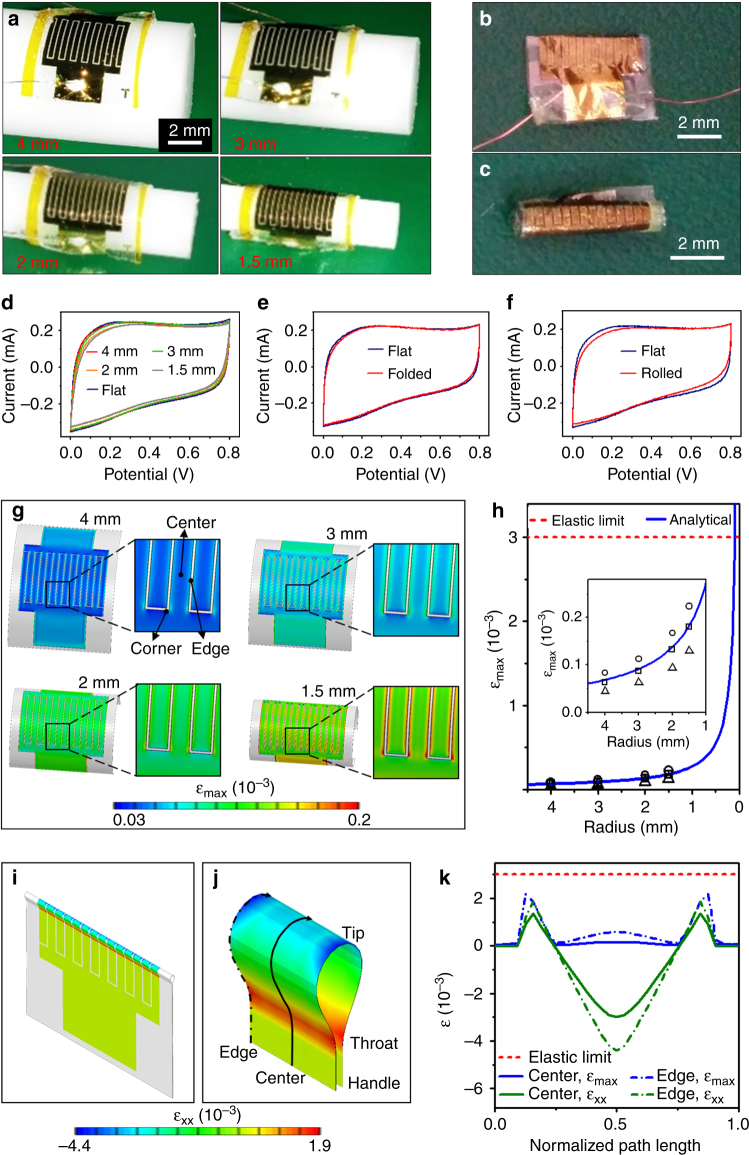
Flexibility and mechanical stability of the SWCNT MSCs. Digital photographs of **a** bent (bending radius = 1.5–4 mm), **b** folded, and **c** rolled MSCs (scale bar = 200 mm). Comparison of CV curves of undeformed (flat) MSCs with those of MSCs **d** bent around rods with radii of 1.5–4 mm, **e** folded, and **f** rolled into a tube with a diameter of ~1.2 mm. FEA results of **g**, the maximum (first principal) strain *ɛ*_max_ in the Au layer of MSCs bent around rods with radii of 1.5–4 mm, **h** *ɛ*_max_ vs. bending radius (the inset shows a magnified view; triangular, rectangular, and circular data points represent *ɛ*_max_ at the center, edge, and corner of an interdigital finger, respectively), **i** *ɛ*_xx_ in the Au layer of a folded MSC, **j** magnified plot of *ɛ*_xx_ in the Au layer of interdigital finger of a folded MSC (the *x*-direction is along the arrowed curves), and **k** variation in *ɛ*_max_ and *ɛ*_xx_ along the paths shown in **j** (the arrowed solid and dash-dot curves represent one-half of the total path distance along the center and edge of the electrode finger, respectively)

Figure 4 illustrates the flexibility of SWCNT MSCs on an ultrathin PI substrate. This is demonstrated by the unique capability to undergo excessive bending, folding, and rolling without significant degradation of the electrical performance. Figure 4a shows SWCNT MSCs on a PI substrate bent around rods with radii of 1.5–4 mm. The CV performance versus bending radius at a scan rate of 10 V s^–1^ (Fig. 4d) shows insignificant changes in the electrical performance due to bending. In fact, the microdevice bent around a rod with a radius of 1.5 mm demonstrates ~91% capacity retention (Fig. S9). Figure 4b shows a microdevice folded along its middle axis without the formation of a crease. Although the significant deformation decreased the device area by 50%, the CV response was not affected (Fig. 4e), indicating that the folded microdevice retained ~100% of its capacity, which is illustrative of excellent mechanical stability. Rolling the SWCNT MSC on a PI substrate into a tube with a diameter of ~1.2 mm (Fig. 4c) significantly decreased the device area, but did not affect the electrical performance (Fig. 4f)—the rolled microdevice showed ~92% capacitance retention (determined by the hysteresis area of the CV response of the rolled and flat (undeformed) devices). These findings indicate that folding and rolling are effective ways to increase the areal capacitance of these microdevices without degrading the electrical performance. Thus, the configuration of the SWCNT MSCs on ultrathin PI substrates can be easily altered by bending, folding, and rolling, according to the device area and energy-storage capacity requirements of a specific application.

Figure 4g shows the distribution of maximum (first principal) strain *ε*_max_ at the top surfaces of Au current collectors of microdevices bent around rods of different radii. For each bending radius, *ε*_max_ was obtained at the center, edge, and corner of the interdigital fingers (Fig. 4g). As shown in Figure 4h, the *ε*_max_ at the center, edge, and corner of the interdigital fingers (triangular, rectangular, and circular data points, respectively) is less than the yield strain (~0.003) by at least an order of magnitude. An analytical model (Supplementary Note 1) was developed to predict the dependence of the maximum bending strain *ε*_b_ on the bending radius. The analytical results are in good agreement with the FEA results. For SWCNT MSCs rolled 2.5 times around an ~1.2 mm roll diameter, which may also be considered as an extreme case of bending, the analytical model yields *ε*_b_ = 5.29 × 10^–4^, which is significantly below the yield strain. Therefore, the deformation due to bending and rolling is elastic and reversible. Because an irreversible crease caused by folding may result in excessive strain localization, which could significantly affect the electronic performance, the MSC was folded without creating a crease by sequential bending, aligning, and pressing (Fig. S10). In the last step (pressing), surface adhesion resulted in partial bonding of the opposed PDMS surfaces of the MSC, leaving the free-standing part to deform into a shape resembling a tennis racket^45,46^. In the experiments and corresponding FEA models, the lengths of the free-standing parts are equal to ~725 µm. Figure 4i, j show the distribution of strain in the *x*-direction *ε*_xx_ in the Au layer of the folded MSC and a folded interdigital finger, respectively. The *x*-direction is along the arrowed curves shown in Fig. 4j and is also indicated in the undeformed device shown in Fig. S10a. The distribution of *ε*_xx_ near the folding site (Fig. 4j) shows that the tip of the racket is under compression, while both the throat and handle of the racket are under tension. Along the arrowed edge and center paths (the curves in Fig. 4j show one-half of the total path, which has a length of 967 µm), the maximum tensile and compressive *ε*_xx_ strains at the finger edge are approximately 1.9 × 10^–3^ and –4.4 × 10^–3^, respectively, while at the finger center, these values are approximately 1.4 × 10^–3^ and –3 × 10^–3^, respectively (Fig. 4k). Away from the folding site, the strain in the Au layer is almost zero due to the absence of bending. The highest *ε*_max_ in the Au layer is ~2.2 × 10^–3^ (Fig. 4k), which is less than the yield strain (the normalized path length was obtained by dividing the *x*-distance by the total path length of 967 μm). In all of the aforementioned FEA simulations, the strain in the Au interdigital fingers is below the yield strain, indicating that deformation is fully reversible (elastic). Interestingly, although the FEA predicts higher strains in folded fingers than bent and rolled fingers, the folded MSC demonstrates higher capacity retention (~100%) compared with the bent and rolled MSCs (Fig. 4d–f). This seemingly contradictory finding is likely due to the strain in the Au contact pads of the bent and rolled MSCs. The leads were electrically connected to the contact pads with silver paste (typically ~100-µm-thick), which, upon curing, increased the local thickness by approximately fivefold. Consequently, the bending strain in the silver paint increased significantly, resulting in a decrease in the capacity retention, as observed in Figure 4d,f. (FEA does not take into account the increased strain in the silver paste.) In comparison, the Au pads in the folded MSC are almost strain free (Fig. 4i). Therefore, the folded MSC shows uncompromised capacity retention. More robust electrical connections between the leads and Au contact pads may further improve the overall flexibility of the microdevice. The ultrathin structure and intrinsic high flexibility of the PI substrate and excellent mechanical stability of the SWCNT network endow the SWCNT MSCs with exceptional flexibility and surface mounting capability, enabling these microdevices to be integrated into various flexible and/or stretchable chips for applications in roll-up electronics (e.g., displays and TVs), electronic paper, smart sensors, or even wearable electronics.

## Conclusions

SWCNT MSCs were fabricated on free-standing ultrathin PI substrates by combining conventional lithography and mechanical peel-off techniques. The SWCNTs were spray-coated onto the PI substrate to form a mechanically stable and highly conductive SWCNT network without the use of any organic binders, conductive additives, or polymer separators, which are often used in commercial supercapacitors. This enhanced the device performance because of the easy access of electrolyte ions to the active material. The high power density (1125 W cm^–3^) of the SWCNT MSCs is attributed to the high conductivity and easily accessible surface area of the spray-deposited SWCNT network and the short distance between the interdigital fingers of the in-plane electrodes. These findings provide a plausible solution to microscale energy storage in many applications where electrolytic capacitors fail to provide sufficient energy density. Moreover, the present microdevices demonstrate excellent electrochemical stability, as evidenced by their ~96% capacity retention even after 100,000 charge/discharge cycles. This unique capability is very important when compared with microbatteries, the relatively short lifetime of which is a major limitation in most applications. The long life of the present microdevices is especially important when combined with energy harvesters to produce on-chip self-powered systems. The exceptional flexibility of SWCNT MSCs, demonstrated by bending, folding, and rolling experiments and numerical analysis, paves the way for potential applications of these all-solid-state microdevices as flexible energy-storage systems in portable, stretchable, and wearable electronic devices.

## Electronic supplementary material

Highly flexible, foldable and rollable microsupercapacitors on an ultrathin polyimide substrate with high power density
