# Supplementary material for: Highly flexible, foldable, and rollable microsupercapacitors on an ultrathin polyimide substrate with high power density
Source: Microsyst Nanoeng. 2018 Jul 30;4:16. doi: 10.1038/s41378-018-0016-3 (PMC6220169; doi:10.1038/s41378-018-0016-3)
Supplement: Supplementary file 1 — Highly flexible, foldable and rollable microsupercapacitors on an ultrathin polyimide substrate with high power density [file 41378_2018_16_MOESM1_ESM.docx]

**Highly flexible, foldable and rollable microsupercapacitors on an ultrathin polyimide substrate with high power density**

Juan Pu^1,2^, Xiaohong Wang^1,*^, Renxiao Xu^2^, Sixing Xu^1^ and Kyriakos Komvopoulos^2,*^

^1^Department of Microelectronics and Nanoelectronics, Tsinghua University, Beijing 100084, China

^1^Institute of Microelectronics ,Tsinghua University, Beijing 100084, China

^1^Tsinghua National Laboratory for Information Science and Technology, Beijing 100084, China

^2^Department of Mechanical Engineering, University of California, Berkeley, California 94720, USA

**Supplementary Information**

__________________________

*Corresponding authors:

Prof. X. Wang: Tel.: +86-01062798432, Fax: +86-1062771130, E-mail: [wxh-ime@tsinghua.edu.cn](mailto:wxh-ime@tsinghua.edu.cn)

Prof. K. Komvopoulos: Tel.: +1-510-642-2563, Fax: +1-510-642-5539, E-mail: kyriakos@me.berkeley.edu

**Table S1.** Performance comparison of MSCs developed in this work and previous studies.

| Electrode material | Electrode thickness (μm) | Electrolyte | Volumetric capacitance  (F cm^–3^) | Energy density  (mWh cm^–3^) | Power density  (W cm^–3^) | Time constant (ms) | Ref. |
| --- | --- | --- | --- | --- | --- | --- | --- |
| SWCNTs | 0.14 | PVA–H_3_PO_4_ | 18 (0.5 V s^–1^) | 1.6 | 1125 | 1 | This work |
| RGO-CNT (9:1) | 6 | 3 M KCl | 6.1 (0.01 V s^–1^) | ~0.68 | ~77 | 4.8 | [1] |
| OLC | 7 | 1 M Et_4_NBF_4_/  anhydrous C_4_H_6_O_3_ | 1.3 (1 V s^–1^) | ~1.4^[a]^ | ~250^[a]^ | 26 | [2] |
| SWCNT | ~3.5 | 3.6 M H_2_SO_4_ | 1.04 | 1.73^[b]^ | 568^[b]^ | 7 | [3] |
| aMP | 5 | PVA–H_3_PO_4_ | 12 (0.1 V s^–1^) | 1.6 | 90 | 14 | [4] |
| G/CNTCs | 10-20 | 1 M Na_2_SO_4_ | 1.1 (0.2 V s^–1^) | 0.16 | 30 | 1.8 | [5] |
| LSG | 7.6 | PVA–H_3_PO_4_ | 2.0 (1 V s^-1^) | 0.25 | 65 | 19 | [6] |
| ERGO | 20 | 25% KOH | 0.24 (40 µAcm^–2^) | --- | --- | 0.17 | [7] |
| Porous diamond foam | ~2.6 | 3 M NaClO_4_ | 2.3 | 1^[b]^ | 202^[b]^ | 3.16 | [8] |

RGO = reduced graphene oxide; OLC = onion-like carbon; aMP = activated mesophase pitch; G/CNTCs = graphene/carbon nanotube carpets; LSG = laser-scribed graphene; ERGO = electrochemically reduced graphene oxide.

^[a]^Estimated from Ragone plots from the literature

^[b]^Estimated from the energy and power densities of electrodes given in the literature (E_cell_ = ¼ E_electrodes_, P_cell_ = ¼ P_electrodes_)


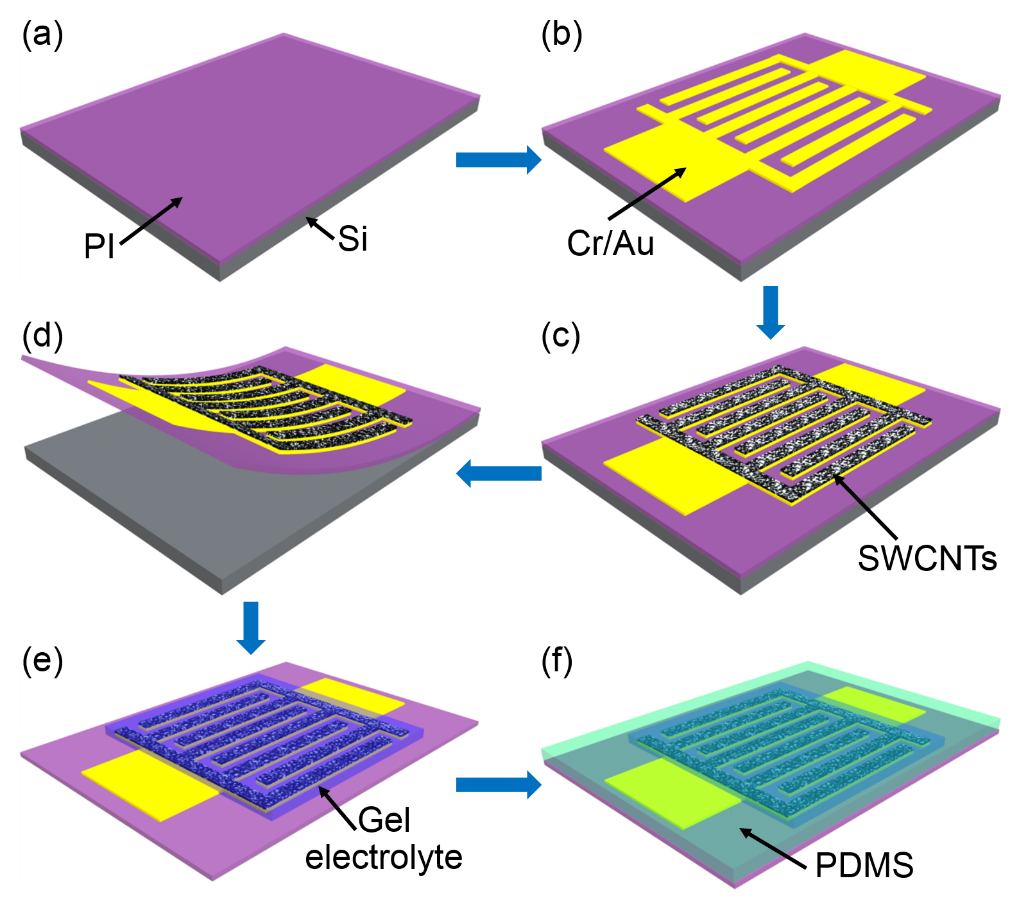


**Figure S1**. Fabrication process of flexible SWCNT MSCs on an ultrathin PI substrate: (a) spin coating of a PI layer on a Si(100) wafer, (b) evaporation and micropatterning of a Cr/Au bilayer, (c) spray deposition and micropatterning of interdigital SWCNT electrodes, (d) peeling-off of the device from the wafer, (e) coating of the interdigital SWCNT electrodes with a gel electrolyte, and (f) encapsulation of the device with PDMS.


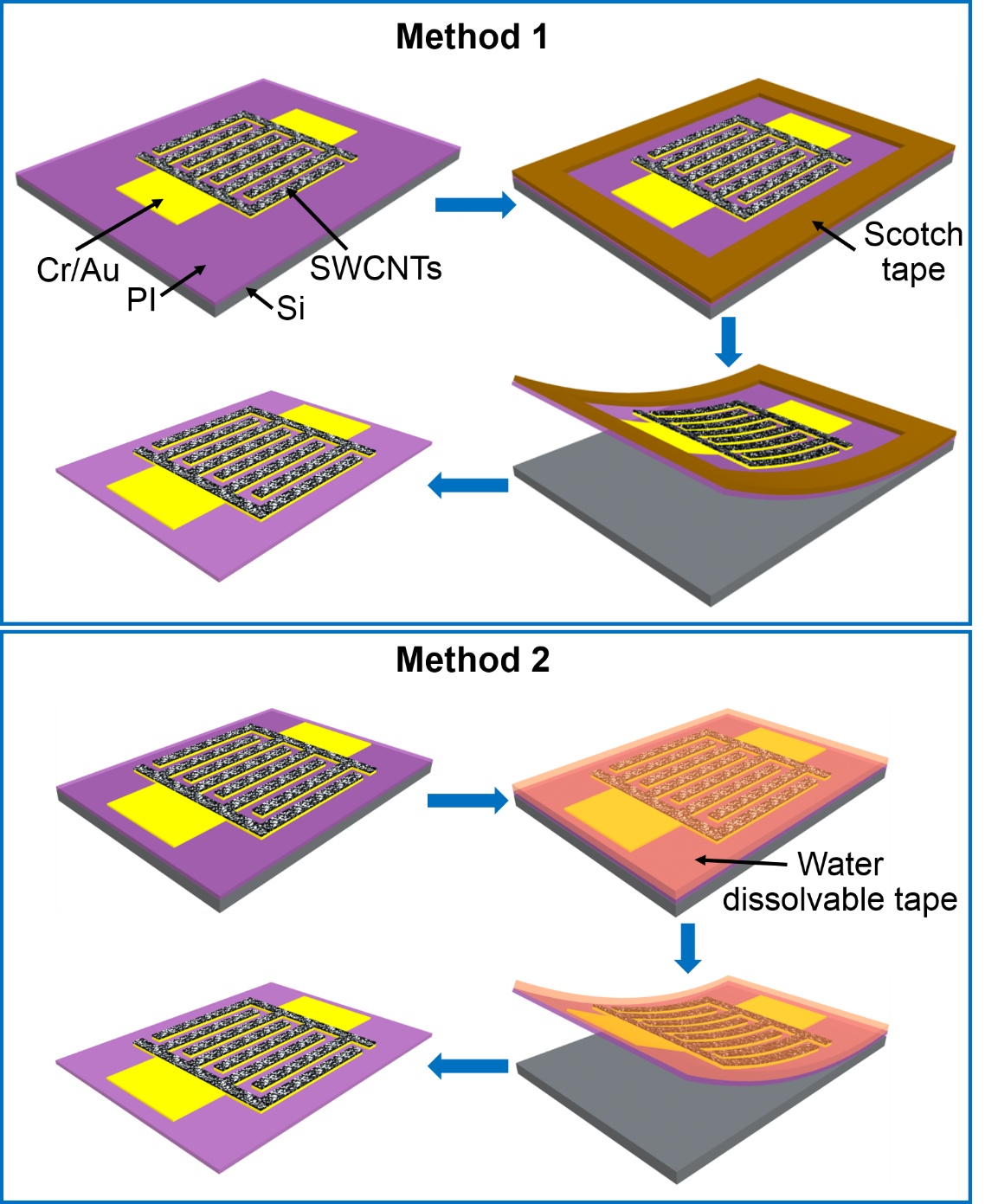


**Figure S2**. Peel-off processes of the PI layer with the attached microdevice from the Si substrate.

*Method 1*: A scotch tape is applied onto the PI surface around the device area and removed after peeling off the device from the Si substrate.

*Method 2*: A water dissolvable 3M tape is applied onto the device surface, dissolved in DI water, and then removed after peeling off the device from the Si substrate.


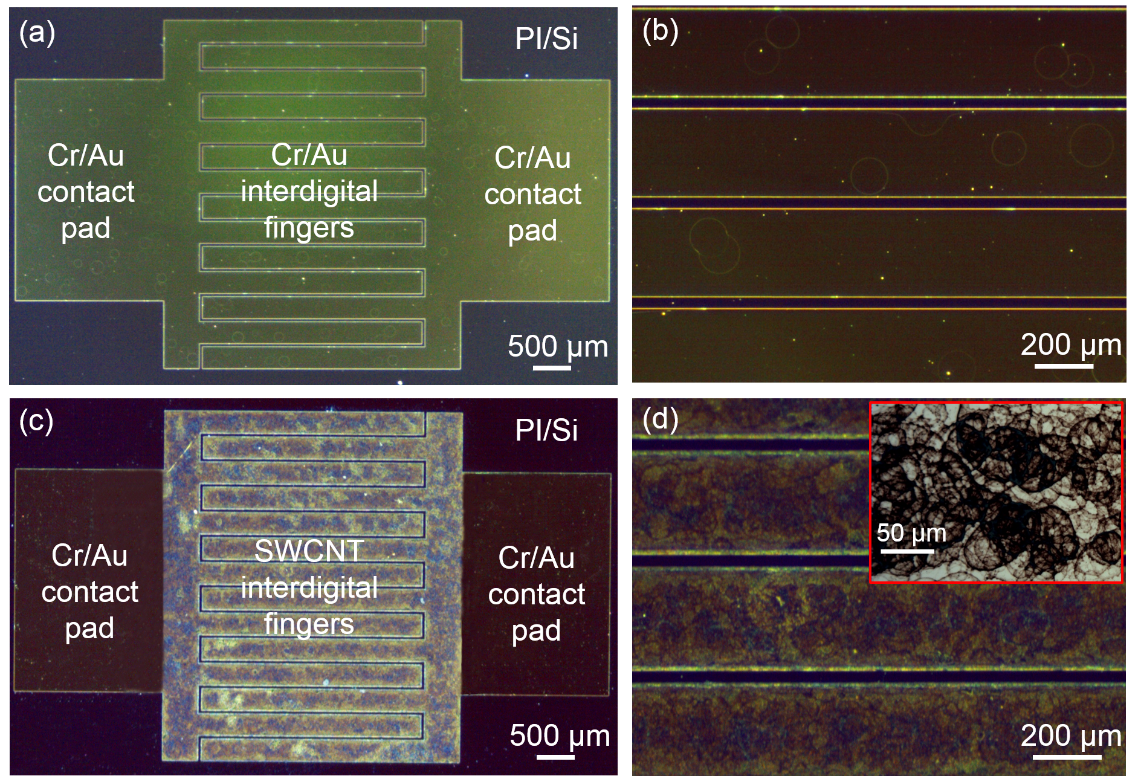


**Figure S3**. Optical microscope images of (a) patterned Cr/Au bilayer deposited onto a PI-coated Si substrate, (b) interdigital Cr/Au fingers, (c) patterned SWCNTs on top of the Cr/Au bilayer, and (d) SWCNT interdigital fingers. The inset in (d) shows a close-up view of the SWCNT electrode.

**Figure S4**. Cross-sectional SEM images of (a) Cr/Au+SWCNTs and (b) Cr/Au layers deposited on a PI layer.


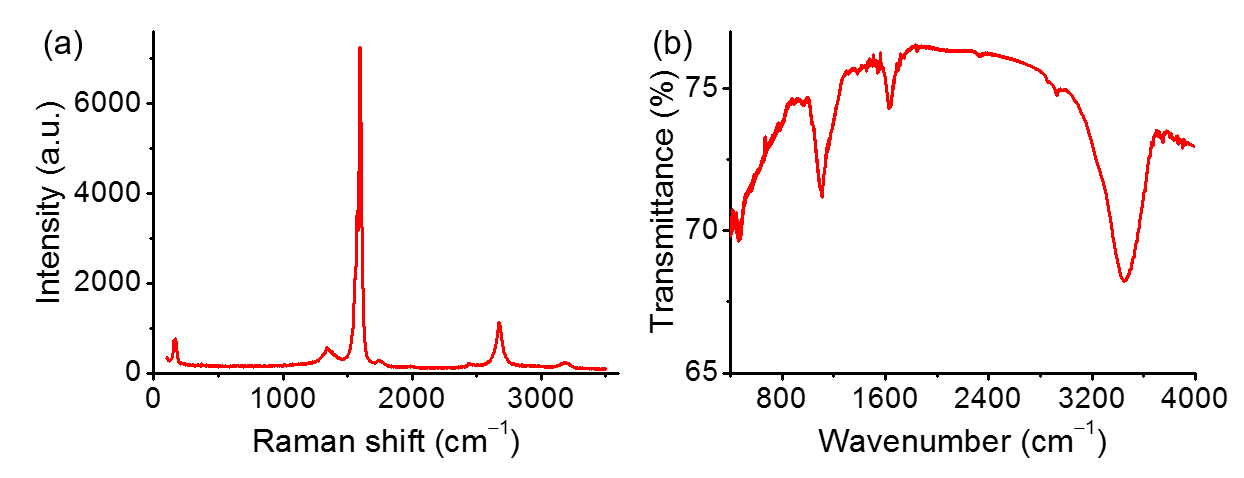


**Figure S5**. (a) Raman and (b) FTIR spectra of SWCNTs. For the Raman spectrum shown in (a), *I*_D_/*I*_G_ = 0.078. The peaks at 1112, 1635, and 3448 cm^–1^ in the FTIR spectrum shown in (b) are assigned to –CH bending [10,11], CNT back bone [12], and carboxylate –OH stretching [11], respectively.

**Figure S6**. Double edges of bilayer lift-off photoresist (PR), indicated by red dashed lines, after UV exposure and development, showing the formation of an undercut surface assisting the lift-off process.


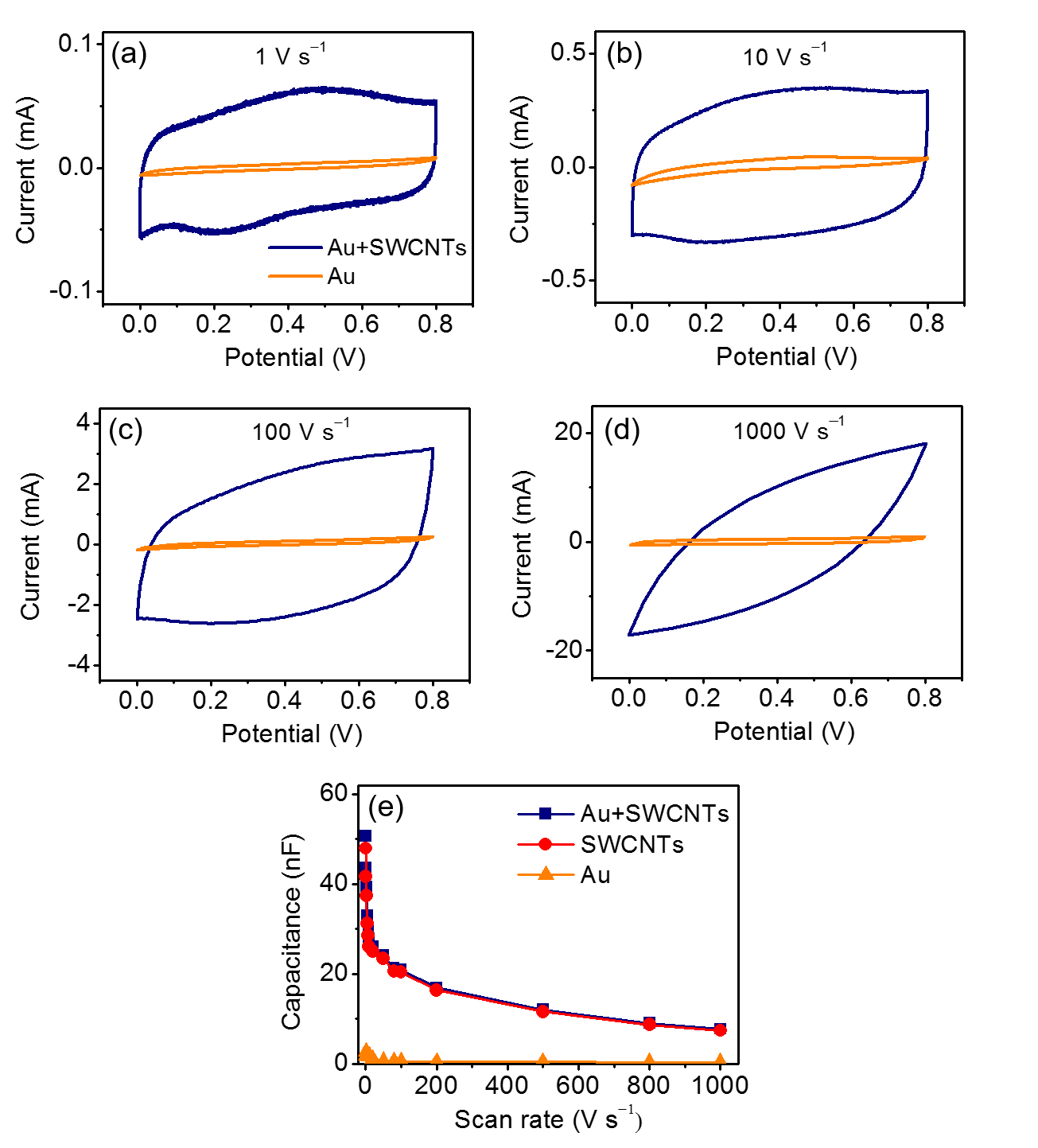


**Figure S7**. Comparison of CV curves of bare Au and Au+SWCNTs electrodes for a scan rate equal to (a) 1, (b) 10, (c) 100, and (d) 1000 V s^–1^, and (e) capacitance of Au+SWCNTs, pure SWCNTs, and bare Au electrodes versus scan rate. The capacitance of SWCNTs electrodes was obtained by subtracting the capacitance of Au electrodes from the total capacitance of Au+SWCNTs electrodes.

**Figure S8**. GCD curve of a SWCNT MSC for a current density of 10 nA cm^–2^.


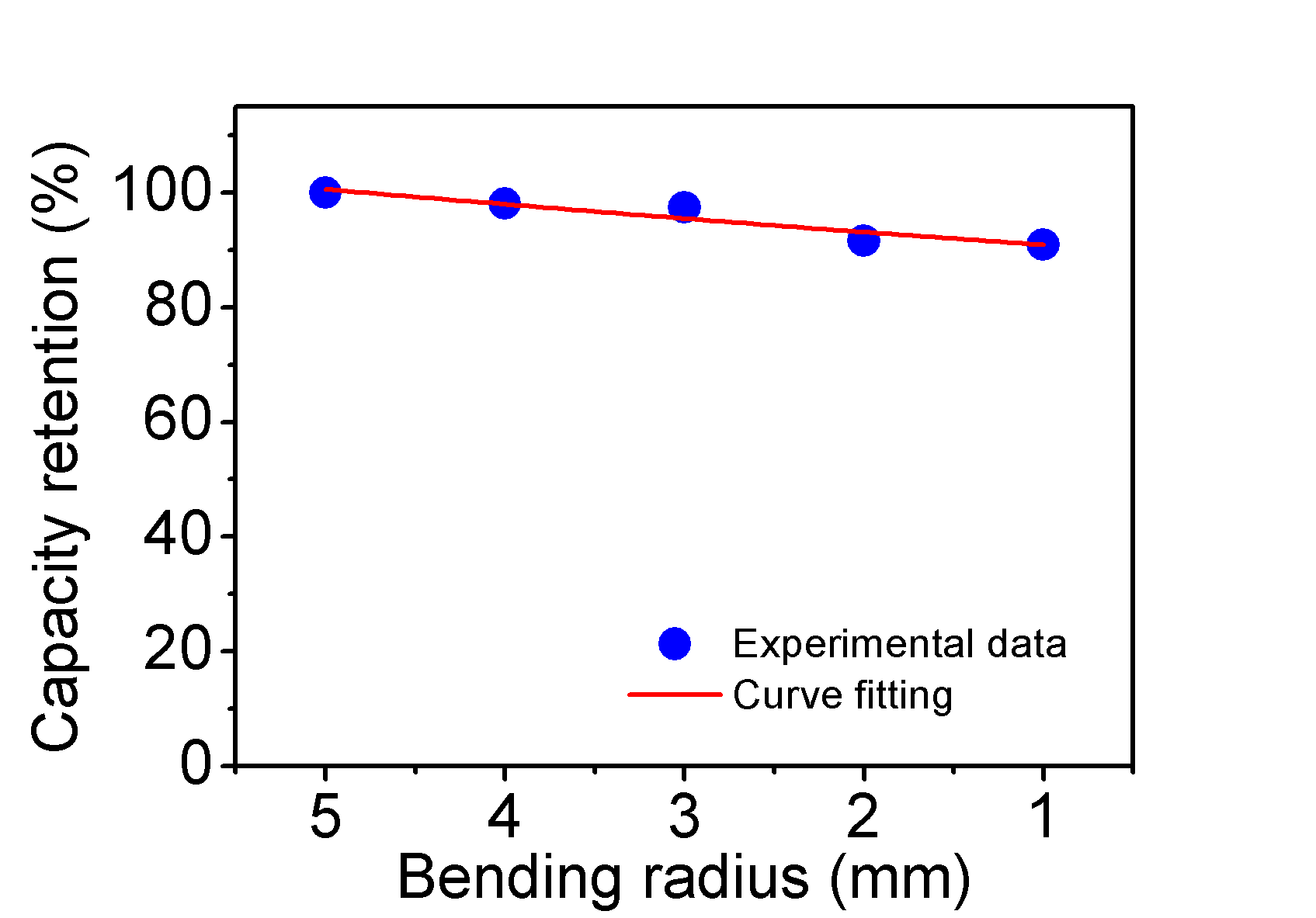


**Figure S9**. Capacity retention of flexible SWCNT MSC versus bending radius.

**
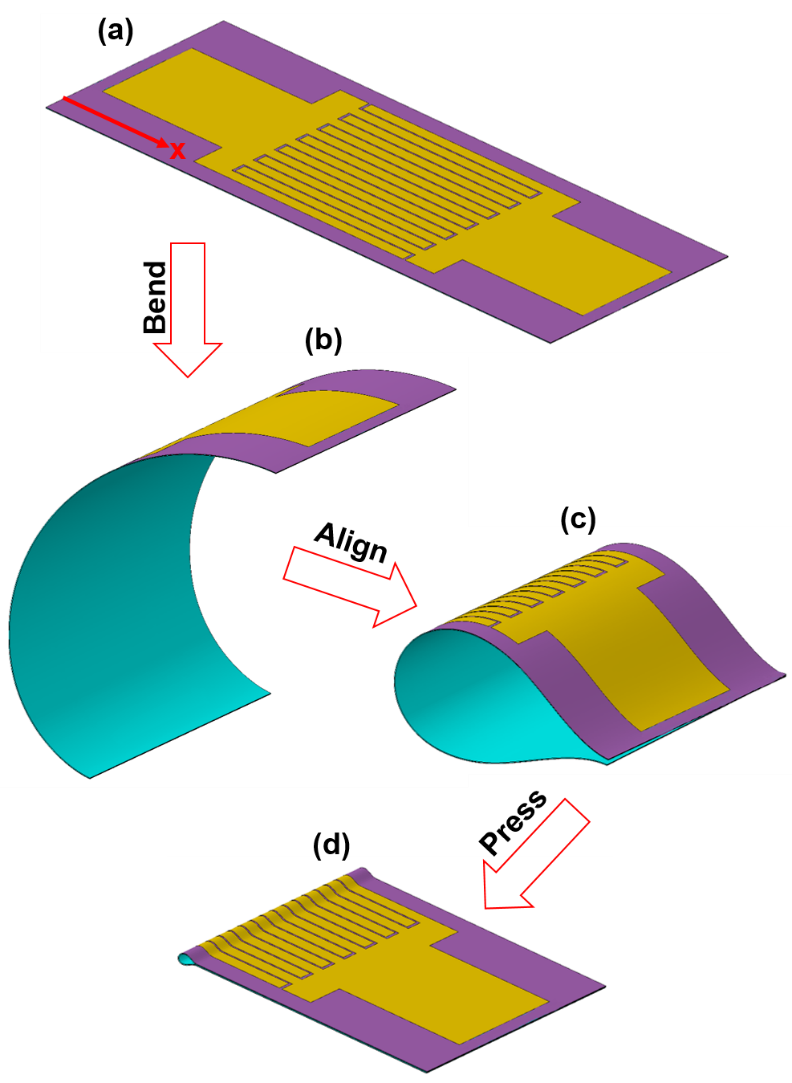
**

**Figure S10**. Schematic of the folding process of a SWCNT MSC.


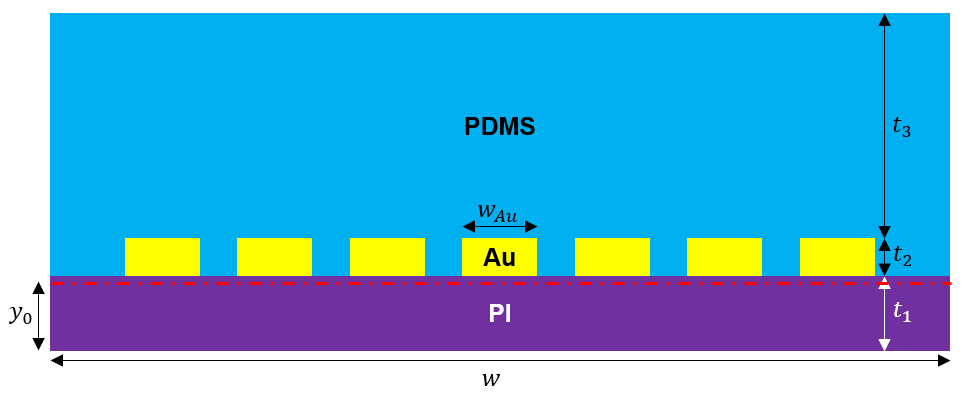


**Figure S11**. A simplified cross-sectional geometry of SWCNT MSCs.

**Note 1. Dependence of maximum bending strain on bending radius**

Fig. S11 shows a simplified cross-sectional MSC geometry. The bottom layer consists of PI ($E_{\mathrm{PI}}=2.5 \mathrm{GPa}$, $\nu_{\mathrm{PI}}=0.34$, $w_{\mathrm{PI}}=w=5720 \mu m$, ${t_{1}=t}_{\mathrm{PI}}=1.3 \mu m$). There are $n=14$ interdigital fingers consisting of Au ($E_{\mathrm{Au}}=78 \mathrm{GPa}$, $\nu_{\mathrm{Au}}=0.44$) with a rectangular cross-section ($w_{\mathrm{Au}}=300 \mu m$, ${t_{2}=t}_{\mathrm{Au}}=130 \mathrm{nm}$) deposited on top of the PI layer. The encapsulating PDMS layer ($E_{\mathrm{PDMS}}=1.1 \mathrm{MPa}$, $\nu_{\mathrm{PDMS}}=0.49$, $t_{3}=t_{\mathrm{PDMS}}=10 \mu m$) spontaneously fills the gaps between fingers. The effective modulus of the first (PI), second (Au), and third (PDMS) layer is

$E_{1}=E_{\mathrm{PI}}$

$$E_{2}=\left( \frac{nw_{\mathrm{Au}}}{w} \right)E_{\mathrm{Au}}+\left( \frac{w-nw_{\mathrm{Au}}}{w} \right)E_{\mathrm{PDMS}}$$

$E_{3}=E_{\mathrm{PDMS}}$ (S1)

The distance $y_{0}$between the neutral axis and the bottom PI surface is given by

$y_{0}=\frac{\sum_{i=1}^{3} \left[ E_{i}t_{i}\left( -\frac{t_{i}}{2}+\sum_{j=1}^{i} t_{j} \right) \right]}{\sum_{i=1}^{3} E_{i}t_{i}}$ (S2)

Substitution of dimensions and material properties in Eq. (S2) yields $y_{0}=$ 1.155 µm.

The neutral axis is denoted by a dash-dot red line in Fig. S11. The maximum bending strain $\varepsilon_{b}$ in the Au metal is given by

$\varepsilon_{b}=\left( t_{1}+t_{2}-y_{0} \right)/R$ (S3)

where $R$ is the bending radius.

When the MSC is wrapped around a rod of radius $R_{0}$, the bending radius is $R=R_{0}+y_{0}$.

The shape of the deformed MSC is modeled as an Archimedes spiral of the form

$r\left( \theta\right)=R_{0}+t(\theta/2\pi)$ (S4)

where $t=\sum_{i=1}^{3} t_{i}$ is the total thickness of the MSC and $r(\theta)$ is the radius at an angle $\theta$.

The spiral length $L$ can be calculated by integrating $r(\theta)$ from 0 to a given rotation angle $\Theta$, i.e.,

$L=\int_{0}^{\Theta} r(\theta)d\theta=R_{0}\Theta+\frac{t}{4\pi}\Theta^{2}$ (S5)

Because $\varepsilon_{b}$ corresponds to the smallest bending radius in the MSC, the smallest bending radius for $L=8400 \mu m$ and 2.5 rounds ($i.e., \Theta=5\pi$) is$R=R_{0}=\left[ L-\left( t/{4\pi} \right)\Theta^{2} \right]/\Theta\approx520.5 \mu m$.

Substitution of $R=520.5 \mu m$ into Eq. (S3) gives $\varepsilon_{b}=$5.29 × 10^–4^.

**References**

1. Beidaghi M, Wang C. Micro‐supercapacitors based on interdigital electrodes of reduced graphene oxide and carbon nanotube composites with ultrahigh power handling performance. *Adv. Funct. Mater.* 2012; **22**: 4501–4510.

2. Pech D, Brunet M, Durou H, *et al*. Ultrahigh-power micrometre-sized supercapacitors based on onion-like carbon. *Nature Nanotechnol.* 2010; **5**: 651–654.

3. Laszczyk KU, Kobashi K, Sakurai S, *et al*. Lithographically integrated microsupercapacitors for compact, high performance, and designable energy circuits. *Adv. Energy Mater.* 2015; **5**: 1500741.

4. Huang HC, Chung CJ, Hsieh CT, *et al*. Laser fabrication of all-solid-state microsupercapacitors with ultrahigh energy and power based on hierarchical pore carbon. *Nano Energy* 2016; **21**: 90–105.

5. Lin J, Zhang C, Yan Z, *et al*. 3-dimensional graphene carbon nanotube carpet-based microsupercapacitors with high electrochemical performance. *Nano Lett.* 2013; **13**: 72–78.

6. El-Kady MF, Kaner RB. Scalable fabrication of high-power graphene micro-supercapacitors for flexible and on-chip energy storage. *Nature Commun.* 2013; **4**: 1475.

7. Sheng K, Sun Y, Li C, *et al.* Ultrahigh-rate supercapacitors based on electrochemically reduced graphene oxide for ac line-filtering. *Sci. Rep.* 2012; **2**: 247.

8. Gao F, Wolfer MT, Nebel CE. Highly porous diamond foam as a thin-film micro-supercapacitor material. *Carbon* 2014; **80**: 833–840.

9. Čeponkus J, Smilga AP, Rumskaitė I, *et al*. Infrared absorption spectroscopy of functionalized single-walled carbon nanotubes. *Chemija* 2013; **24**: 9–19.

10. Esfandiary E, Valiani A, Hashemibeni B, *et al*. The evaluation of toxicity of carbon nanotubes on the human adipose-derived-stem cells *in-vitro*. *Adv. Biomed. Res.* 2014; **3**: 40.

11. Singh DK, Iyer PK, Giri PK. Functionalization of carbon nanotubes and study of its optical and structural properties. *Nanotrends* 2008; **4**: 55–58.
